# What Are the Common Themes of Physician Resilience? A Meta-Synthesis of Qualitative Studies

**DOI:** 10.3390/ijerph19010469

**Published:** 2022-01-01

**Authors:** Nurhanis Syazni Roslan, Muhamad Saiful Bahri Yusoff, Karen Morgan, Asrenee Ab Razak, Nor Izzah Ahmad Shauki

**Affiliations:** 1 Department of Medical Education, School of Medical Sciences, Universiti Sains Malaysia, Kubang Kerian 16150, Malaysia; msaiful_bahri@usm.my; 2 Perdana University-Royal College of Surgeons in Ireland School of Medicine, Kuala Lumpur 50490, Malaysia; karenmorgan@perdanauniversity.edu.my; 3 Department of Health Psychology, Royal College of Surgeons in Ireland, D02 YN77 Dublin, Ireland; 4 Department of Psychiatry, School of Medical Sciences, Universiti Sains Malaysia and Hospital USM, Universiti Sains Malaysia, Kubang Kerian 16150, Malaysia; asrenee@usm.my; 5 Institute for Health Systems Research, National Institutes of Health, Ministry of Health, Shah Alam 40170, Malaysia; drizzah@moh.gov.my

**Keywords:** resilience, physicians wellbeing, hardiness, grit, engagement, mental health, burnout, healthcare, meta-synthesis

## Abstract

In the practice of medicine, resilience has gained attention as on of the ways to address burnout. Qualitative studies have explored the concept of physician resilience in several contexts. However, individual qualitative studies have limited generalizability, making it difficult to understand the resilience concept in a wider context. This study aims to develop a concept of resilience in the context of physicians’ experience through a meta-synthesis of relevant qualitative studies. Using a predetermined search strategy, we identified nine qualitative studies among 450 participants that reported themes of resilience in developed and developing countries, various specialties, and stages of training. We utilized the meta-ethnography method to generate themes and a line-of-argument synthesis. We identified six key themes of resilience: tenacity, resources, reflective ability, coping skills, control, and growth. The line-of-argument synthesis identified resilient physicians as individuals who are determined in their undertakings, have control in their professional lives, reflect on adversity, utilize adaptive coping strategies, and believe that adversity provides an opportunity for growth. Resilient physicians are supported by individual and organizational resources that include nurturing work culture, teamwork, and support from the medical community and at home. Our findings suggest that resilience in physicians is dynamic and must be supported not only by physician-directed interventions but also by organization-directed interventions.

## 1. Introduction

The complex system of patient care requires physicians to endure long hours, keep electronic medical records, manage administrative work burdens, and deal with public health crises such as the coronavirus 2019 (COVID-19) pandemic [1]. A longitudinal study showed that physicians were twice as likely to experience burnout compared with the general population, with an increasing trend between 2011 and 2014 [2]. A recent systematic review of 182 studies across 45 countries reported the prevalence of burnout ranging from 0% to 80.5% [3]. Furthermore, such worrying prevalence was not limited to burnout but also other mental health issues, such as depression and anxiety [4,5]. A recent meta-analysis also reported a high prevalence of burnout, depression, anxiety, stress, and post-traumatic stress disorder during the recent COVID-19 pandemic [6].

Several interventions have been developed to improve mental health issues among physicians, which can be broadly grouped into physician-directed and organizational-directed [1]. Although the root of the problem lies mainly within healthcare systems and organization-directed interventions have been shown to be more effective in reducing burnout, interventions have been primarily focused on physicians [1,7]. An increasingly studied physician-directed intervention is resiliency training [8]. The concept of resilience expands the discourse on physician’ mental health by highlighting the role of empowering human strength in surviving adversity—that is, positive psychology [9,10].

The word resilience is derived from the Latin “resilire,” which means to leap back or recoil [11,12]. The Oxford Dictionary defined resilience as “the ability of people or things to recover quickly after something unpleasant such as shock or injury” [13]. Studies on human resilience have stemmed from child psychology, where researchers conducted phenomenological observations on children who seemed to have suffered little damage compared with the significant adversity they experienced in childhood [14]. Children that achieved better than the expected outcomes were identified as resilient or stress-resistant [9]. Resilience studies then evolved into three waves of inquiry and began to include a greater number of study populations, such as socioeconomically challenged immigrants, teenagers with single-parent roles, and genocide survivors [14,15]. The first wave characterized the attributes, support systems, and situational enablers of resilient individuals, which included self-determination, faith, creativity, happiness, and excellence [16,17]. The second wave expanded the literature by exploring how such qualities or factors contributed to the development of resilience [17,18]. A central theory in this wave, the resiliency model, proposes that disruption and reintegration play a role in nurturing resilient qualities in individuals [15]. The third wave of inquiry explored the force within individuals that drives them to seek strength and excellence. Resilience was described as the “protective and vulnerable forces that exist at different levels—community, culture, family, and the individual” [19]. A systematic review of general practitioners identified several enablers of resilience at various levels, such as having a sense of purpose (individuals), having control over work matters (within work), and lifestyle (beyond work) [20].

Resilience in physicians has been increasingly studied following Zwack and Schweitzer’s article, “If every fifth physician is affected by burnout, what about the other four? Resilience strategies of experienced physicians” [21]. Quantitative studies using generic resilience scales reported an inverse correlation with mental health indicators, such as burnout, depression, and stress [22,23]. However, a growing number of qualitative studies have examined the concept of resilience as it is perceived in various specialties [24], types of practice [25], and training levels [26]. Such studies are consistent with the contextualized nature of these different settings, which may highlight a different set of resilience themes. However, because the findings of individual qualitative studies are not usually generalizable, they cannot be applied to inform a wide range of practices, policies, or strategies that address physicians’ wellbeing [27,28]. A pragmatic approach to addressing the knowledge generated by these individual qualitative studies is meta-synthesis. Meta-synthesis is an interpretive integration of related qualitative studies, which is more than the sum of its parts and provides novel interpretations of an event or experience [29]. An example is a previous meta-synthesis that identified four common themes of resilience across 21 resilience scales in various populations: commitment to deal with adversity, control, resourcefulness, and growth [30]. Building on this conceptual framework, the present study aims to develop a conceptualization of the resilience construct in the context of physicians through a synthesis of the findings from relevant qualitative studies.

## 2. Methods

### 2.1. Study Design

We utilized a meta-synthesis study design based on a protocol registered in the International Prospective Register of Systematic Review (PROSPERO) [31]. In exploring areas with little known information, meta-synthesis can be viewed as an inductive method that facilitates knowledge or theory development [27,32]. In frequently studied areas, meta-synthesis can be a deductive method used to aggregate findings from individual qualitative studies on a similar topic of interest and that share a methodological rigor similar to a quantitative equivalent of meta-analysis [33].

In this study, we adopted the meta-ethnographic method, which involved a systematic comparison, the translation of one study into other studies (without reducing the original meaning of the data in the primary study), and synthesizing the translation in conceptualizing the resilience construct in the context of physicians’ experience [34,35].

### 2.2. Search Strategy and Selection Criteria

We followed the seven-step process shown in Table 1.

## 3. Results

**Figure 1:**
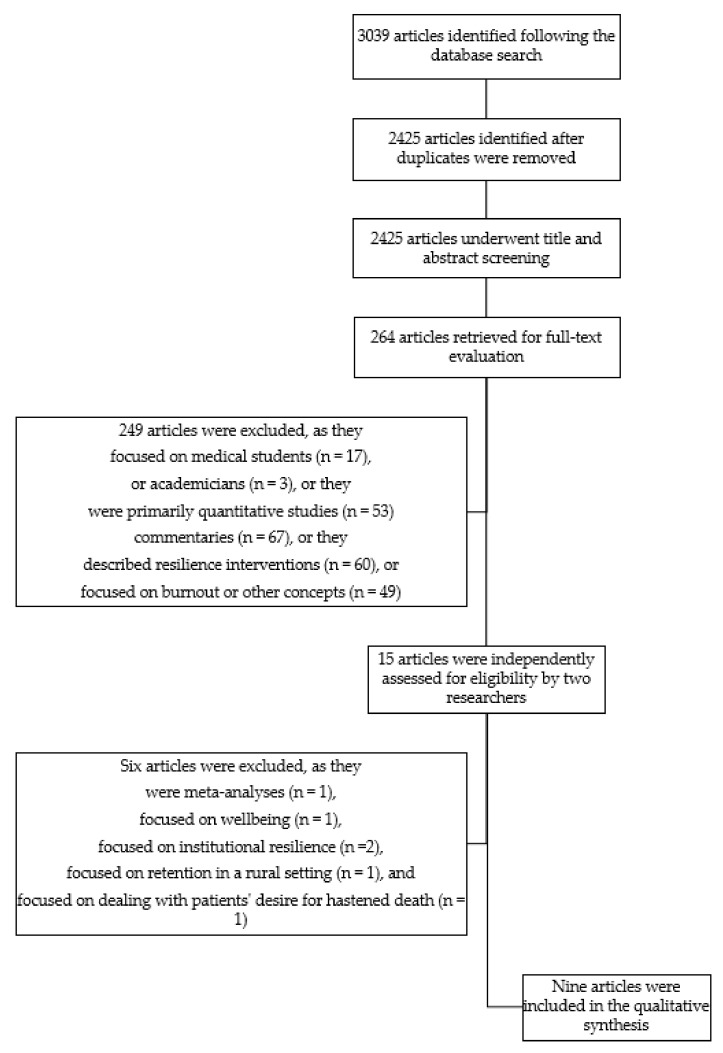
Flow chart summarising the search strategy and results.

Our search from 1 September 2018 to 28 February 2019 generated a total of 3039 hits (Figure 1). In screening the abstracts, we removed 614 duplicate studies and a further 2161 articles that did not meet the inclusion criteria. The excluded studies were mainly concerned with resilience related to medical-related disasters, such as earthquakes and resilience in psychiatric patients and allied healthcare professionals, or they examined related concepts, such as mindfulness and wellbeing. We retrieved 264 studies for full-text evaluation and excluded a further 249. Most of these studies were removed because of the study design (i.e., quantitative, interventional, or commentary), sample (i.e., medical students or academicians), or because they examined related constructs (i.e., burnout). Following independent reviews, two researchers agreed that nine studies should be included, and five should be removed. There was a discrepancy in the decision to include one article. This discrepancy was reviewed by a third researcher who suggested its exclusion, as the study focused on enablers of physicians working in underserved populations rather than resilience. Therefore, nine studies were included in the final synthesis.

### 3.1. Study Descriptions

The final nine studies selected for the synthesis reflected the conceptualization of resilience in physicians in Australia, Canada, Germany, South Africa, United Kingdom, and the United States of America. Details of the included studies are summarized in Table 2. All studies were published between 2007 and 2018 and, in total, included data collected from 450 participants. The studied contexts were general practice (*n* = 3), remote areas (*n* = 2), obstetrics and gynecology (*n* = 1), intensive care (*n* = 1), residency *(n* = 1), and internship (*n* = 1).

The methods used in the selected studies were as follows: in-depth interviews (IDI); nominal group technique (NGT); focus group discussion (FGD); and free text response. The approaches utilized were grounded theory (*n* = 4), mixed methods (*n* = 2), open inquiry (*n* = 1), and inductive (*n* = 1). One study did not specify the research approach. Regarding qualitative grading [36], four studies were graded as excellent, three studies as good, and two studies as fair. The fair grade was mainly due to the context of the studies, which might have limited their generalizability to a wider context, a lack of explanation in data collection, or in the construction of themes.

### 3.2. Themes of Physician Resilience

The derived themes are summarized in Table 3. From 82 themes identified in the selected studies, we formed 11 subthemes and six final themes, which were tenacity, resource, control, coping, reflective ability, and growth. The subthemes and final themes were inducted from the original themes, which we define in the following paragraphs.

#### 3.2.1. Theme 1: Tenacity

Tenacity is defined as the determination to continue one’s actions [43]. This theme incorporates two subthemes: aspiration and commitment.

##### Aspiration

A study conducted with obstetrics and gynecology residents proposed a conceptual model of resilience in the form of a growing tree. Aspiration was reflected by the sun, which sustained and stimulated the professional growth of physicians [24]. Most studies proposed that deriving deep meaning from work or patient contact made physicians engage with their professional calling despite its struggles and challenges [24,25,38,39,40].

*“I can’t think of anything else that I would rather be doing.”* [39]

*“...what is more intimate that being in the room and delivering somebody’s baby…we appreciate how intimate that moment is, and how special it is to get to be a part of it.”* [24].

Some studies also highlighted that believing it was the right thing to do made them consider challenges as motivations to be more resilient in patient care 40.

*“There are patients that you see that you think, “Oh dear, it’s so-and-so again” and you say to yourself, that this is a person who’s got rights, who is doing their best to live their life by their own values, their own circumstances, and you start to see the good in them, you start to see their achievements, their essential humanity.”* [40].

##### Commitment

Several studies proposed that “showing interest in the person behind the symptom “empathy” and “connecting with patients” were important elements of resilience. These often led to good doctor–patient relationships and patients’ appreciation, which was a continuous source of resilience in physicians [21,24,38,41].

*“My favorite, which happens when nobody’s looking, is when the patient says, ‘Can I come back and see you? You’re so good. Thank you so much. That was the first time somebody’s explained this to me.”* [24].

A study conducted with residents in Germany proposed that staying committed required physicians to embrace the reality of medical practice. Avoiding wishful thinking prevented physicians from discouragement and resentment about being over-extended at work [21].

#### 3.2.2. Theme 2: Resources

All studies in the synthesis proposed that resilience development was not derived only from the individuals but also from their surroundings in the workplace and at home. Resources emerged as an important element of physician resilience because they support individual capacity to face adversity. Theme 2 incorporates three subthemes: support, teamwork, and institutional culture.

##### Support

The participants referred to family and friends as effective buffers that counterbalanced their hectic work lives [21,24,26,39]. The participants also proposed the role of “non-medicine people” in providing neutral opinions and in reminding them of their needs, which doctors often feel guilty about fulfilling [21,24].

*“It’s important to find the right balance between self-overestimation and a lack of self-confidence. You need an environment of family and friends who will tell you when you start behaving badly. My wife is my severest critic.”* [21].

##### Teamwork

Most studies also highlighted the role of “teamness” at work, which facilitated heavy workloads, gave feedback, and provided professional input. Working as a team helped to absorb the sources of stress in delivering high-quality patient care. It also fostered resilience at both the individual and organizational levels [21,24,25,26,41].

*“How your day is structured, how everybody works together, and that I think creates a much more resilient work force in terms of the practice but also individually, it reinforces your own resilience.”* [41].

##### Institutional Culture

The studies also highlighted the role of institutional culture in promoting or impeding physician resilience [21,24,25,26,38,39,40,42]. One study found that bureaucratic practice was a significant source of stress that added little to patient care [42].

*“... seeing the patients is a piece of cake, the bureaucracy around seeing them is unbelievable.”* [42].

Another study also pointed out the need for a programmatic approach or a positive culture that promotes resilience among physicians. Such an approach or culture prioritizes feedback, supervision, and coaching, and it provides platforms for the exchange of opinions [21].

*“...the hardest part of residency is you go from 20-plus years of being in school where you get a gold star, you get an A, you get these pats on the back. In clinical work, where these rewards are not always visible, the residents can get discouraged. During residency, ‘This is just the expectation, this is what you’re doing, this is a job’ and there’s no one to be like, ‘Wow, that was a great job you did today.”* [24].

#### 3.2.3. Theme 3: Control

Participants in different studies emphasized that, in addition to tenacity, physicians needed to have control of their professional lives to be more resilient. The theme of control incorporated several subthemes: understanding professional boundaries at work, acknowledging one’s own limitations, and striving for work–life balance.

##### Professional Boundaries

Participants emphasized that a clear understanding of the professional boundaries could provide prophylactic and symptomatic relief because physicians consciously understand the line between endless expectations and professional standards [21,39,40,41]. Understanding job expectations and actual responsibilities also helped physicians to set limits and prevent overwork.

*“Knowing what your role is and sticking to that, I suppose being assertive with other disciplines.”* [41].

Understanding one’s own professional identity and boundaries also fostered a better sense of control and assisted physicians’ decision-making in their practice [40,41].

##### Acknowledging One’s Own Limitations

Participants emphasized that it is important for physicians to be self-aware of having realistic expectations, accepting personal limitations, and forgiving themselves for making errors [21,26,38,39]. Participants also commended institutions that dealt with error management positively through meetings or circles, as it made it easier for them to move on despite their guilt and turn the anguish of medical errors into a learning experience [21].

*“It takes a certain amount of humility to be able to say ‘you know what, I can’t figure this out by myself’ … and there’s nothing wrong with that.”* [39].

##### Work–Life Balance

Most studies also highlighted that the concept of work–life balance was an important element of physician resilience. Participants consistently highlighted how leisure time or long-time hobbies not only helped to alleviate their stress and burnout but also helped to focus on work from a better perspective [21,25,26,39,42].

*“I have to look after myself, and I can’t believe how much more productive and energetic I am if I pay attention to that piece.”* [39].

Participants also proposed maintaining a work–life balance as a means of self-care in maintaining resilience. Some described ways in which the work structure needed to allow some flexibility for pursuing a work–life balance [25].

#### 3.2.4. Theme 4: Adaptive Coping

Participants mentioned the role of proactively anticipating stress or problems and dealing with them in a timely manner as a means of maintaining resilience [24,41]. Participants also pointed out several adaptive coping strategies, such as prioritizing workload, being organized, and talking about their work stress [21,24,41,42]. In their conceptual model, Winkel et al. (2018) proposed that a coping mechanism expanded physicians’ capacity to grow.

*“I think there’s an element too,... with resilience that you come across the issue and you’re arrogant enough and confident enough that you can come up with a solution to solve it and to cope with whatever issue it is that comes through the door.”* [41].

#### 3.2.5. Theme 5: Reflective Ability

Highlighted among several themes in Zwack and Schweitzer’s (2013) study, reflective ability was found to encourage physicians to consciously think about their professional growth. Being reflective also helped physicians to appreciate positive events that they might have taken for granted but that enhanced their work satisfaction and often became a turning point in self-care [21].

*“I regularly ask myself questions like: Where am I right now? Where do I want to go? What do I find uncongenial? Why am I dissatisfied? What can I do to change that? Another good idea is to do this at a particular time. Ask yourself: Where were the perks last year? Where are they this year?”* [21].

#### 3.2.6. Theme 6: Growth

Four studies proposed that growth was an important theme in physician resilience [21,25,40,41]. Growth was fostered by being flexible and optimistic in facing challenges [41].

*“But it’s also looking at things in a kind of positive light, it’s not being drummed down, it’s looking at it as ‘Well I can solve this’, it’s looking at the cup half full I’d say. It’s ‘I can solve this.’* [41].

Several studies also proposed that engaging with professional development opportunities helped physicians in the face of adversity by maintaining their interest in medicine and improving their professional efficacy [21]. Another study proposed that setting a pragmatic marker of success or acknowledging professional growth, even on a small scale, helped physicians to be more resilient [40].

*“Substance abuse is always a difficult area, because you don’t get many cures. But, if you get someone onto methadone and give them out of goal, that might be a success. If you keep someone alive for 5 years longer than they would have otherwise, you get to judge that as a success as well.”* [40].

### 3.3. Line-of-Argument Synthesis

**Figure 2:**
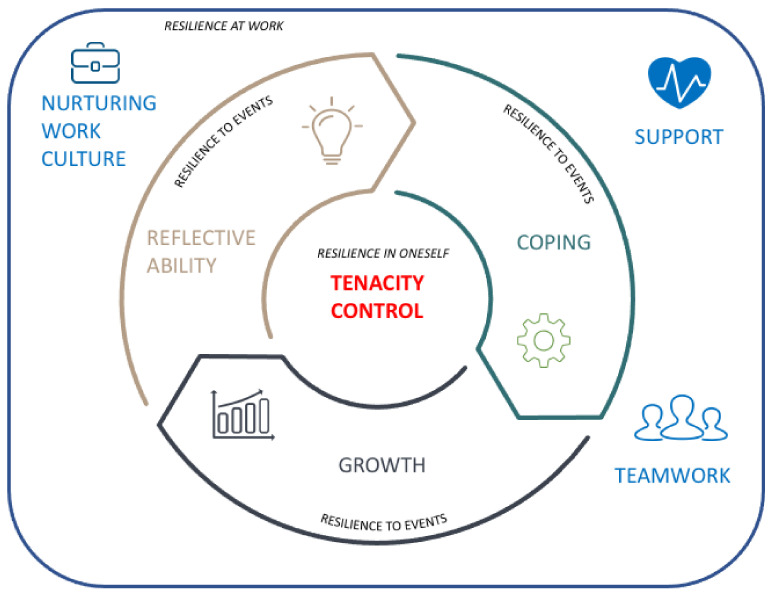
Conceptual model of themes of physician resilience derived from the meta-synthesis performed in this study.

Resilient physicians are tenacious individuals who are determined in their undertakings and have control in their professional lives. In facing adversity, resilient physicians explore the event through their reflective ability, utilize adaptive coping strategies, and remain optimistic that the adversity offers them opportunities for personal and professional growth. Resilient physicians have resources that enable them to deal with adversity through a nurturing work culture, teamwork among colleagues, and support by professional counterparts and at home. These themes form the components of the resilience conceptual model, as shown in Figure 2. After we completed the meta-synthesis, we found a recently published relevant primary study [44]. We were able to incorporate its findings into our synthesis, which suggested that theoretical saturation had been reached.

We also conducted a quality assessment on each theme in the meta-synthesis based on the CERQual Assessment of Confidence [37], as summarized in Table 4. The meta-synthesis explored the common themes of physician resilience in the literature. The primary studies included both Western and non-Western contexts and participants in various career stages and specialties. Of the six derived themes, five were graded as of moderate to high quality, suggesting that the findings were likely to be a reasonable representation of the studied phenomena [37].

## 4. Discussion

The themes generated from the meta-synthesis fit all three waves of resilience inquiry [17]. Some of the themes described qualities in resilient physicians, such as tenacity and growth. Reflective ability, coping skills, and control form the process of resilience development during practice. Aspiration (a subtheme of tenacity) and resources represented the force at the individual and organization levels essential to promote resilience development in physicians. Taken together, the themes proposed a dynamic nature of resilience instead of a stable trait–quality. This is consistent with the American Psychological Association (APA) who define resilience, in general, as “the process of adapting well in the face of adversity, trauma, tragedy, threats, or significant sources of stress” [45].

Tenacity acts as the core of resilience in physicians, similar to the model proposed in the context of obstetrics and gynaecology residents, in which resilience was visualized as a growing tree, and aspiration acts as a sun that drives the tree towards it [24]. Tenacious people have a “stick-to-it-iveness quality” and endure challenges until their goals are achieved [46]. They anticipate obstacles and embrace obstacles as a challenge to deal with and not as a barrier. Most physicians have foreseen the challenges in the profession in medical schools and meet more challenges along with their career. Tenacity conferred a toughening effect to persevere these challenges. A similar theme, hope, was described in a review of nursing resilience, wherein goal orientation gave nurses control, lightened the effects of stress, and engaged them to persevere [47]. While specific quantitative studies on tenacity among physicians are scarce, a study found that passion significantly predicted higher levels of engagement and lower burnout in university students [48]. However, passion is more linked to the grit concept, and this further highlights the overlaps between the grit and resilience concepts [49,50].

Previous literature has construed resilience as “persevering through adversities and returning to a state of internal equilibrium” [51] or “bouncing back stronger following adversities” [52]. Our findings seem to agree with the latter, where engagement with professional development activities and having pragmatic markers of success are described as subthemes in growth [21,40]. This is consistent with most of the professional bodies practices that requires physicians to engage with continuous professional development to cope with the changing needs of patient care [53,54]. Optimism, which forms one of the subthemes of growth, encourages physicians to appraise work challenges as transient, specific, and external (in contrast to constant, pervasive, and internal) [55]. This corresponds to a previous study that demonstrated that optimism predicted resilience against distress [56].

On top of that, the inverse of optimism, deficiency focusing, was found to explain 4% of the variance in the psychological empowerment among US nurses [57].

Consistent with the resiliency model [15], reflective ability is critical in facilitating physicians’ conscious consideration of the outcomes of adversities. This is illustrated in a study that evaluated reflective skills training, which found that residents were more comfortable discussing their mental health problems and learned new strategies to address these challenges [58]. An RCT examining the impact of discussion groups incorporating reflective skills among physicians reported a significant decrease in burnout and an increase of work engagement [59].

The findings also corroborate the theories of stress, burnout, the resiliency model, and the definition from the APA, that posits the centrality of coping in resilience development [15,45,60,61]. When asked on the nature of resilience, many Irish physicians quoted the ability to cope with challenges in the healthcare rather than thriving [62]. The theme is also in keeping with the biological understanding that resilience is developed when an individual encounters and successfully copes with adversity [63]. Researchers have argued that the effectiveness of coping strategies is not universal and depends on the contexts of the adversities [64]. However, several maladaptive coping strategies, such as behavioural disengagement, denial, self-blame, and self-distraction have been consistently shown to correlate with physician’s burnout, depression, anxiety, and stress symptoms [65,66]. Denial coping, often used by physicians in response to heavy workload and difficult patient encounters, was found to increase rather than decrease emotional exhaustion [67]. This supports the role of coping skills training as one of the physician-directed interventions in reducing burnout or enhancing resilience.

Overworking is a common phenomenon in medicine and fatigue was found to have a major impact on job performance, empathy, complex thinking, personal life, and burnout if left unaddressed [68,69]. A study found that most non-consultant doctors came to work despite being unwell, as they failed to find a cover or avoid displeasing teammates [70]. This meta-synthesis made an important insight in which control forms an important element of resilience development in physicians. Setting clear professional boundaries and maintaining an appropriate proximity and distance with colleagues and patients prevents physicians from being caught with unrealistic expectations and disappointment [21]. The theme also highlights the importance of physicians’ acknowledging their own limitations and embracing their own limits positively [71]. Resilient physicians understand mistakes occur in medicine, assess such mistakes objectively instead of emotionally, and seek appropriate help to bridge any gap therebetween [57,62]. Resilient physicians also give an equal focus on nurturing themselves through self-care and work–life balance [24,62,72]. Although personal–professional lifelines are illusory in medicine, the assessed studies proposed that physicians should acknowledge they can be engaged at work and still be connected to their personal life, which often functions as their resources in difficult times [21,26,73]. For example, a study on American and European residents found a dose-response relationship between reading non-medical books and burnout reduction [74]. At present, there is lack of such studies that show the benefits of life outside work in supporting physicians’ resilience. Such studies in different stages of training are desirable to guide us in a more holistic intervention addressing burnout.

Our findings agree with Panagioti et al. (2016) who found that organization plays an important role in mitigating burnout and increasing resilience. Medicine expands at a high rate and demands the ability of physicians to be continuously updated [75]. Studies reported that physicians often have unmet expectations, owing to the discrepancies between learned and workplace competencies [76]. Hence, a formative working environment would foster resilience through adequate learning opportunities, positive error management, supervision, mentoring, and coaching [73]. Up to 81% of residents in Canada reported that their nutritional needs and sleeping facilities while on-call were lacking. The study also found a significant negative correlation between work dissatisfaction and well-being [77]. Hence, workplaces should equip physicians with the basic needs to function effectively, such as facilities and access to physical and mental health care [78].

As burnout is a systemic problem, it is only plausible that it be addressed through a concerted intervention. Although the modern Hippocratic Oath demands the commitment of physicians to attend to their own wellbeing as part of professional abilities to patients [79], studies also proposed that physicians are not well supported, especially when they are faced with conflicts [80]. Despite the inclusion of focus on physician well-being through a culture of wellness and workplace efficiency in the quadruple aim approach [81], there is still an implied expectation that physicians should place their professional commitment over their own welfare [82]. Hence, a central move that requires workplace and training program to include active measures (such as attention to work intensity, permission for emergency or parental leave without fear of adverse consequences, and the reduction of non-physician obligation) would empower physicians to take actions on their well-being [83].

As physicians often struggle with chronic workplace stress, ongoing efforts on primordial and primary intervention at workplace play an important role [79]. Mental health surveillance, for example, would provide a window for early intervention before burnout and other mental health problem develop [84]. Despite concerning mental problem prevalence among physicians at all stages of training, studies reported physicians’ reluctance to seek mental health care due to a lack of time, a preference to manage problems themselves, a lack of access to confidential care, and a belief that colleagues will have less confidence in them [85]. Hence, secondary and tertiary preventions, such as policy to gain access to confidential and affordable health assessment, should be instituted in all workplace and training centres [83].

## 5. Conclusions

In contrast to the component defining hardiness [86], we found that resilience, in the context of physicians, is dynamic and conceptualized by individual qualities, such as tenacity and growth, skills, such as reflective ability, coping, and control mechanisms, and forces at the individual (aspiration) and organizational (resources) level. The strength of the meta-synthesis includes a rigorous study selection and a judicious interpretation of findings across studied context [28]. The findings from the meta-synthesis signals that repetitive explorative studies in the context of physicians yielded a similar framework of resilience. This is shown by the quality assessment, which shows that all of the themes had a moderate-to-high confidence assessment, except for reflective ability. However, reflective ability is supported by resilience theory [15], hence, the overall themes may represent resilience development phenomena in physicians. A possible limitation of the study includes the small number of primary studies that fit the inclusion criteria. However, the findings from a recent study published after the duration of the meta-synthesis produced consistent themes [44], suggesting a theoretical saturation and validity of the line-of-argument synthesis. Given the gravity of the situation, the next wave of research should focus on ways to reduce mental health problems and enhance resilience among physicians. Possible interventions to address different themes of resilience are outlined in Table 5. We again echo the call for resilience intervention to not just be directed just toward physicians, but also toward organizational practice [8].

## Figures and Tables

**Table 1:** Seven-step process used in the meta-ethnography method [34,35].

Steps		Process
1	Getting started	We decided to address the question, “*What are the common themes in physician resilience literature?*”
2	Deciding what is relevant to the research question	Based on the question, we set appropriate search terms, criteria, and databases, as shown below. The search terms were checked and refined by the corresponding author’s librarian.Search terms:“resilience” AND (“doctor” OR “physician” OR “intern” OR “trainee” OR “resident” OR “specialist” OR “consultant”) Criteria: Peer-reviewed journal/article/book/thesis English language Dated from 1980–February 2019 Types of studies grounded theory phenomenology ethnography action research case study mixed method research Participants: medical doctor (ranging from intern to consultant) Exclusion criteria: commentary qualitative or mixed method studies with medical students or shadowing programs qualitative or mixed method studies with academicians Databases: Google Scholar (Citation indexes) SCOPUS (Citation indexes) PubMed, PubMed Central, National Library of Medicine (Subject database) Medline (Subject database) PsycINFO (Subject database)
3	Reading the studies	Based on the search results, NSR and KM reviewed all selected papers independently. Any discrepancies were reviewed by MSBY, and the final agreement was achieved by a consensus.We read the full texts of selected papers and appraised the rigor, credibility, and relevance of the individual papers using an 18-item checklist from the Framework for Assessing Qualitative Evaluations [36].In this step, we began to identify the main themes of the selected papers.
4	Determining how the studies are related	We created a table that included the year of study, participant training stage, sample size, method, and original themes in the primary studies. We then examined the recurring themes across the selected studies.
5	Translating the studies into one another	Using a grid, we systematically compared the themes across the selected papers to identify a range of themes. To preserve the meaning conveyed by the selected papers, we examined the interpretation of the themes in its original term (first order) and checked for reciprocal translation (similar themes) and refutational translation (disconfirming themes). In order to minimize potential biases that could arise from our beliefs and experiences, we spent time in refutational translation to search for disconfirming themes and discussed the interpretations from various perspectives.
6	Synthesizing translations	In this step, we formed overarching themes from the reciprocal themes (second order). Related second-order themes were then merged under a broader theme (third order). These second-order and third-order themes were discussed among all the researchers to examine their congruence with the original themes in the selected studies. As the third-order themes are testable interpretations [35], we assessed our confidence in these themes using the Confidence in the Evidence from Reviews of Qualitative Research (GRADE-CERQual) [37]. We then developed a line of argument in a statement that summarized the common themes in physician resilience.
7	Expressing the synthesis	We formed a framework to explain the line of argument in a comprehensible format for potential audiences, such as clinicians, educationists, and policy makers.

**Table 2:** Summary of studies included in the synthesis.

Authors and Publication Year	Country	Subgroups	Number of Participants	Methods (Approach)	Qualitative Grading *
[38]	United States of America	intensive care unit physicians	14	IDI (grounded theory)	C
[39]	Canada	general practitioners	17	IDI (open inquiry)	B
[40]	Australia	doctors working in challenging areas	15	IDI (grounded theory)	B
[21]	Germany	residents from various specialties	200	IDI (mixed-methods study)	A
[25]	South Africa	health practitioners working in rural areas	29	nominal group technique (not specified)	C
[41]	United Kingdom	general practitioners and health professionals	20	focus group discussion (inductive approach)	A
[26]	United States of America	interns	103	free text response (mixed-methods study)	A
[42]	United Kingdom	general practitioners	34	IDI (modified grounded theory)	A
[24]	United States of America	obstetrics and gynecology residents	18	IDI (grounded theory)	B

* A (excellent), B (good), C (fair), graded based on the Framework for Assessing Qualitative Evaluations [36].

**Table 3:** Summary of themes derived from the meta-synthesis.

Original Themes (First Order)	Subthemes (Second Order)	Final Themes (Third Order)
pride ^1^ valuing physician role ^2^ entering the field ^3^ personal meaning of work ^3^ shared purpose ^5^ value oneself ^7^ aspirations and values ^9^	aspiration	tenacity
empathy ^1^ professionalism ^1^ altruism ^1^ culture ^1^ acceptance and realism ^4^ interest in the person behind the symptom ^4^ tolerant ^7^ connection with patients and work ^9^	commitment	
personal support ^2^ cultivation of relations with family and friends ^4^ support from family, friends or roommates ^6^ support from significant others ^6^ support from family and community ^9^	support	resource
trust/respect ^1^ quest for and cultivation of contact with colleagues ^4^ working in a team ^5^ support from colleague ^6^ good working relationship / teamwork ^7^ relationship with medical community ^9^	teamwork	
resources ^1^ professional support ^2^ organizational support ^3^ institutionalized exchange forums ^4^ supervision, coaching, psychotherapy ^4^ culture of support ^5^ supportive program environment and faculty ^6^ system-level strategies ^8^ programming and culture ^9^	institutional culture	
professional arena ^2^ locus of control ^3^ accepting personal boundaries ^4^ self-demarcation with patients ^4^ self-demarcation with colleagues ^4^ self-discipline in connection with diagnosis and information ^4^ professional boundaries ^7^	professional boundaries	control
ability to detect gaps ^1^ self-awareness ^2^ proactive limitation with the limits of one’s own ^4^ error management ^4^ receiving mental health care ^6^ accept professional limitations ^7^ attention to self ^9^	acknowledging own limitations	
personal arena ^2^ leisure time activity ^4^ limitation of working hours ^4^ ritualized time-out period ^4^ long-time, non-professional field of interest ^4^ prioritization of basic needs ^4^ spirituality ^4^ work-life balance ^5^ time off work, free time, outside interests, social life ^6^ exercising and engaging in self-care ^6^ appreciate humour ^6^ taking leave ^8^	work-life balance	
self-organisation ^4^ talking about job-related stress ^4^ active engagement with the downside of the medical profession ^4^ recognizing when change is necessary ^4^ focus and deal with problems ^7^ using initiatives ^7^ anticipate situations, react and deal ^7^ good organizational skills ^7^ improving efficiency of working day ^8^ personal coping strategies ^8^ effort ^9^	adaptive coping	coping
personal reflection and goal setting ^4^ self-awareness and reflexivity ^4^ creating inner distance by taking an observer perspective ^4^ appreciating the good things ^4^	reflective ability	reflective ability
pragmatic markers of success ^3^ cultivation of one’s own professionalism ^4^ opportunities for growth ^5^ optimism ^7^ flexible and adaptable ^7^ confidence ^7^	growth	growth

primary studies: ^1^ [38], ^2^ [39], ^3^ [40], ^4^ [21], ^5^ [25], ^6^ [26], ^7^ [41], ^8^ [42], ^9^ [24].

**Table 4:** Quality assessment of the themes derived from the meta-synthesis.

Themes that Emerged from Meta-Synthesis	Assessment of Methodological Limitations	Assessment of Relevance	Assessment of Coherence	Assessment of Adequacy	Overall Assessment of Confidence *
tenacity ^1–5,7,9^	moderate concerns (two studies with moderate limitations)	no concern	minor concerns (data consistent across studies)	moderate concerns (three studies with thin data)	moderate
resource ^1–9^					high
control ^1–9^					moderate
coping ^4,7–9^	minor concerns (one study with minor limitations)	moderate concerns (possible partial relevance-developing countries context)	moderate concerns (data consistent across some studies)	minor concerns (one study with thin data)	moderate
reflective ability ^4^	no concern	substantial concern (partial relevance as described on one context)	moderate concerns (data consistent within one studies)	no concern	low
growth ^3–5,7^	minor concerns (one study with minor limitations)	no concern	moderate concerns (data consistent across some studies)	minor concerns (one study with thin data)	moderate

* Confidence in the Evidence from Reviews of Qualitative Research (GRADE-CERQual) [37]. Primary studies: ^1^ [38], ^2^ [39], ^3^ [40], ^4^ [21], ^5^ [25], ^6^ [26], ^7^ [41], ^8^ [42], ^9^ [24].

**Table 5:** Potential physician- and organization-directed interventions to address different themes of resilience [1,7,8,81,82].

Themes Derived from Meta-Synthesis	Potential Physician-Directed Interventions	Potential Organization-Directed Interventions
Tenacity	Medical student selection Informed specialty choices Mindfulness training Balint groups	Optimal patient contact time Opportunities to attend professional development programs
Resource	Training to improve team communication, conflict resolution and making effective requests to administrators	Duty hour limits Provision of support staff to reduce clerical burdens Provision of adequate on-call facilities Providing a safe and ergonomic working environment
Control	Training in breaking bad news Grief counselling	Engagement with physicians on work structures and requirements Flexible work schedules Part-time posts Incentivized exercise program Permission for appropriate medical, emergency, or parental leave without fear of adverse impact to employment Mental health surveillance Confidential access to mental health services
Coping	Coping skills training Stress management training	Reducing unnecessary bureaucracy Provision of a system that accepts feedback from physicians
Reflective ability	Balint groups Mindfulness training Reflective skills training	Regular debriefing sessions
Growth	Balint groups	Mentoring or coaching programs Feedback practice Provision of support for professional developments

## Data Availability

The data presented in this study are available on request from the corresponding author.
